# Arrhythmogenic right ventricular cardiomyopathy with left ventricular noncompaction: A phenotypic overlap with high arrhythmic risk

**DOI:** 10.1016/j.hrcr.2026.06.034

**Published:** 2026-07-03

**Authors:** Mario José Mayorga Duarte, Christopher Kaleb Romero Ríos, Darwin Rizo Cortedano, Andrés Zamora Díaz, Byron Larios Alemán, Faustino Jesús Silva Centeno

**Affiliations:** 1 Department of Cardiology, Hospital Militar Escuela “Dr. Alejandro Dávila Bolaños,” Managua, Nicaragua; 2 School Of Medicine, Hospital Militar Escuela “Dr. Alejandro Dávila Bolaños,” Managua, Nicaragua; 3 Department of Imagenology, Hospital Militar Escuela “Dr. Alejandro Dávila Bolaños,” Managua, Nicaragua

**Keywords:** Arrhythmogenic cardiomyopathy, Left ventricular noncompaction, Cardiac magnetic resonance, Left bundle branch area pacing, Cardiac resynchronization therapy defibrillator

**Key Teaching Points**

• Arrhythmogenic cardiomyopathy extends beyond right ventricular disease and includes biventricular and left-dominant phenotypes. Recognition of this broader spectrum, as emphasized by the Padua criteria, is essential for accurate diagnosis and clinical decision-making.
• Phenotypic overlap between arrhythmogenic cardiomyopathy and left ventricular noncompaction may reflect a shared desmosomal genetic substrate, particularly involving desmoplakin variants, and should prompt careful multimodality evaluation and cascade family screening. Cardiac magnetic resonance plays a central role in identifying structural abnormalities and myocardial tissue characterization.
• Cardiac sarcoidosis should be carefully excluded when intramyocardial septal late gadolinium enhancement is present. Absence of extracardiac manifestations and the presence of biventricular fibro-fatty changes support an arrhythmogenic cardiomyopathy diagnosis when fluorine-18 fluorodeoxyglucose positron emission tomography is unavailable.
• A low burden of premature ventricular contractions does not exclude high arrhythmic risk. Quantitative tools, such as the arrhythmogenic right ventricular cardiomyopathy Risk Calculator (Cadrin–Tourigny model), provide individualized 5-year estimates and should complement guideline-based criteria when deciding on primary-prevention defibrillator therapy.
• The presence of non-sustained ventricular tachycardia, structural abnormalities, and conduction delays justifies early consideration of advanced device therapy. In young patients requiring ventricular pacing, physiologic conduction-system pacing through left bundle branch area pacing combined with cardiac resynchronization therapy with defibrillator offers sudden death protection while mitigating pacing-induced cardiomyopathy.

## Introduction

Arrhythmogenic cardiomyopathy (ACM) is an inherited myocardial disease characterized by progressive fibro-fatty replacement of the myocardium, predisposing to ventricular arrhythmias and an increased risk of sudden cardiac death, particularly in young individuals and athletes. Its estimated prevalence ranges from 1:1000 to 1:5000 in the general population, and it accounts for a substantial proportion of sudden cardiac death cases in young adults.^1^^,^^2^ Although traditionally described as a right ventricular (RV) disease, growing evidence has expanded its definition to a broader spectrum that includes left ventricular (LV) and biventricular involvement.^3^^,^^4^

Recent advances in diagnostic frameworks, particularly the 2020 Padua criteria, have emphasized the heterogeneity of ACM and the importance of cardiac magnetic resonance (CMR) for identifying structural and tissue abnormalities.^3^^,^^5^^,^^6^ In this context, the coexistence of ACM with other myocardial phenotypes, such as LV noncompaction (LVNC), represents a rare and poorly characterized overlap that may pose diagnostic and risk stratification challenges.^4^^,^^7^ We present a case of ACM with biventricular involvement and an LVNC phenotype, highlighting its complex presentation and arrhythmic implications.

## Case report

We present a 34-year-old male patient with no relevant past medical history or family history of sudden cardiac death, who presented with progressive exertional dyspnea and recurrent presyncope during physical activity.

**Figure 1:**
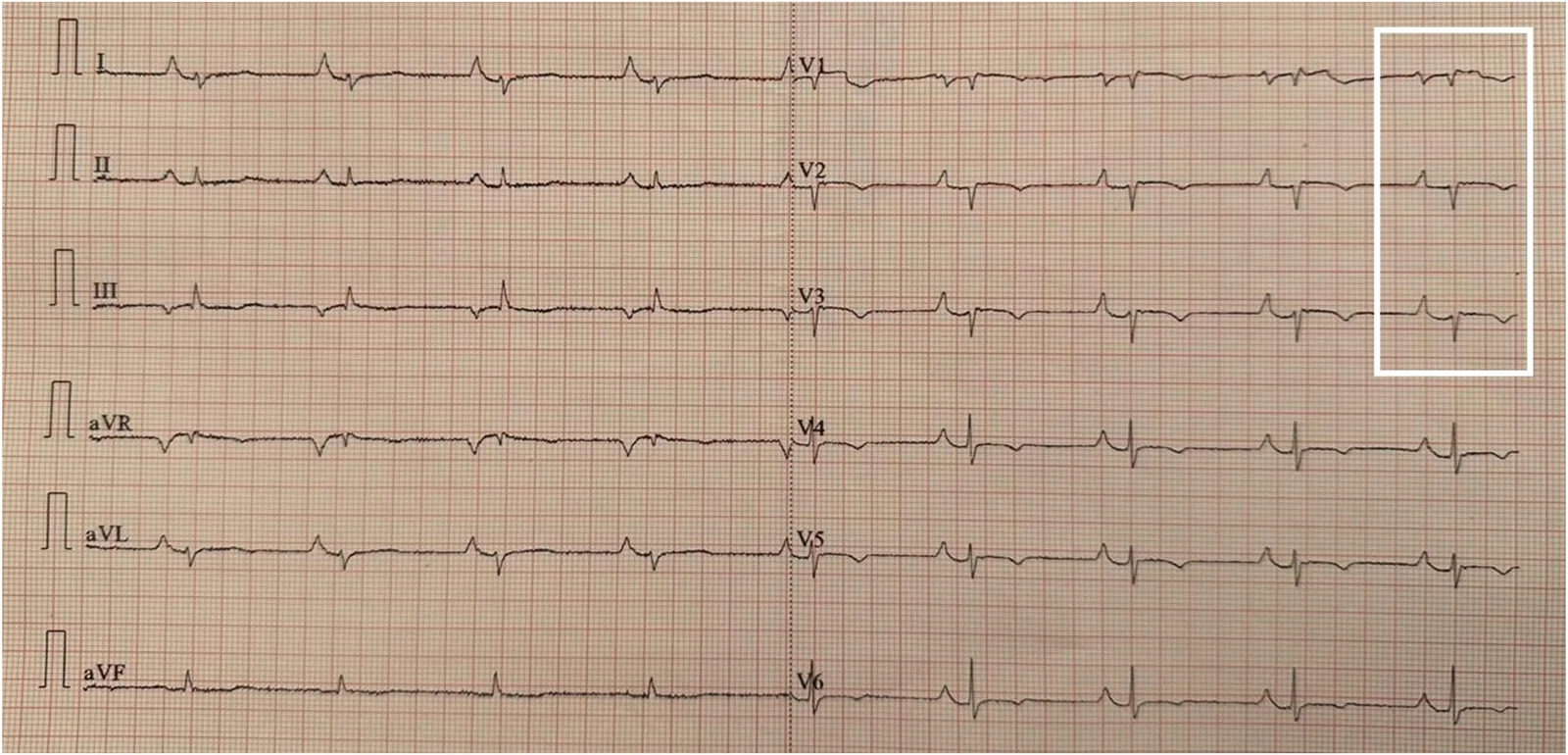
Electrocardiogram. 12-lead electrocardiogram showing sinus rhythm, with T-wave inversion in the right precordial leads (V1–V3) (*white box*), suggestive of right ventricular (RV) involvement in arrhythmogenic cardiomyopathy.

Upon initial evaluation, the physical examination revealed no relevant abnormalities, with vital signs within normal limits and no signs of systemic congestion or hemodynamic compromise. A 12-lead electrocardiogram demonstrated sinus rhythm with a prolonged PR interval and ventricular repolarization abnormalities characterized by T-wave inversion in the right precordial leads (V1-V3), findings suggestive of RV structural involvement (Figure 1).

**Figure 2:**
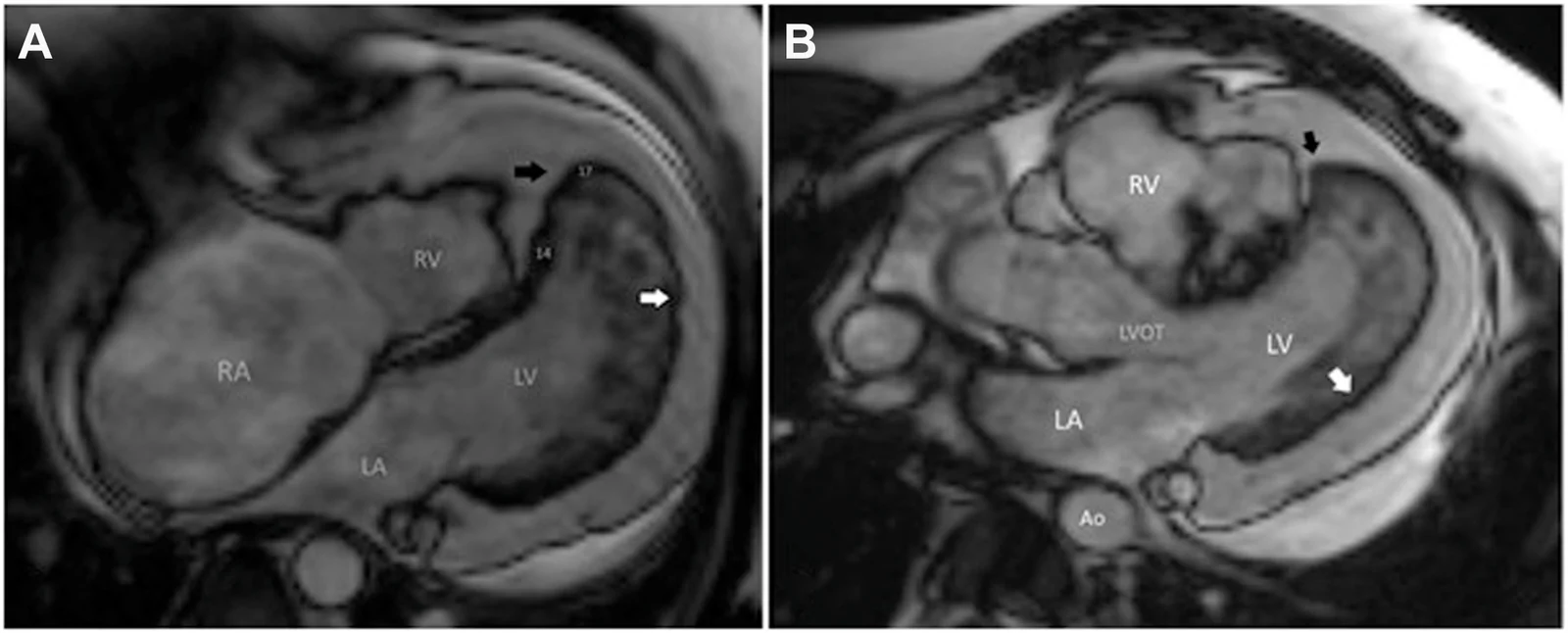
Left ventricular noncompaction (CMR). CMR steady-state free precession (SSFP) cine imaging demonstrating the left ventricle (LV) with a significant increase in trabeculations (*white arrow*). **A:** Long axis, 4-chamber view; **B:** Long axis, 3-chamber view. The apex (*black arrow*) has a curve that points to the right, with a course anterior to the apex of the right ventricle (RV). The left ventricular outflow tract (LVOT) is preserved as well as the aorta (Ao). Furthermore, the apical septal segment (segment 8) and the apex (segment 9) are not compacted (shown in A). LA = left atrium.

**Figure 3:**
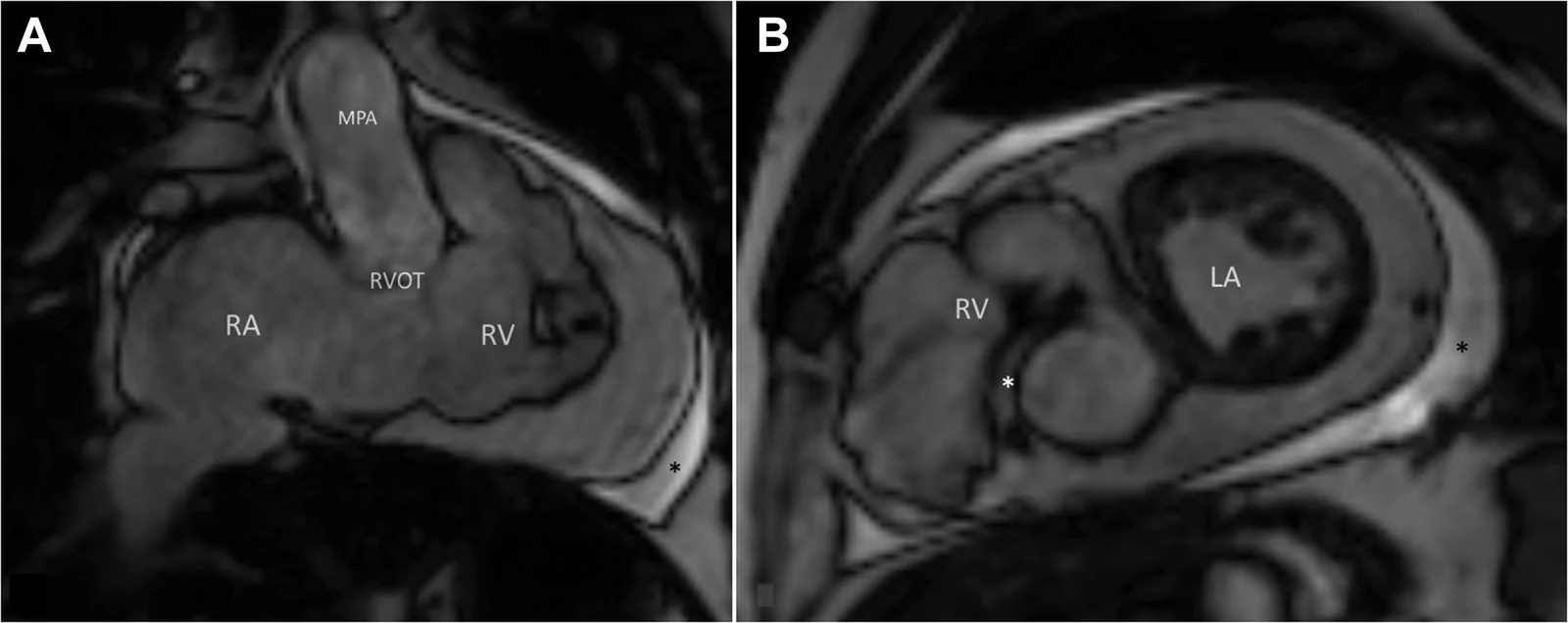
Right ventricular structural abnormalities (CMR). CMR, SSFP sequence. **A:** Right 2-chamber long-axis view; **B:** Short-axis view. The right ventricle (RV) demonstrates severe structural remodeling with irregular, trabeculated walls and bilobed sacculations involving the free wall and right ventricular outflow tract (RVOT). Marked right atrial (RA) enlargement and mild pericardial effusion (∗) are also observed. MPA = main pulmonary artery.

Given the persistence of symptoms and the electrocardiographic findings, CMR imaging was performed for structural characterization, which revealed biventricular involvement. The LV showed a preserved ejection fraction (LVEF: 56.3%), with a significant increase in trabeculations and noncompacted segments at the apical level, highlighting a noncompacted-to-compacted ratio of 3.5, consistent with LVNC (Figure 2). Conversely, the RV presented moderate systolic dysfunction with an ejection fraction of 32% (RVEF: 32%), associated with structural remodeling featuring global hypokinesia, irregular walls, prominent trabeculations, and sacculation in the free wall and outflow tract, findings highly suggestive of ACM (Figure 3).

**Figure 4:**
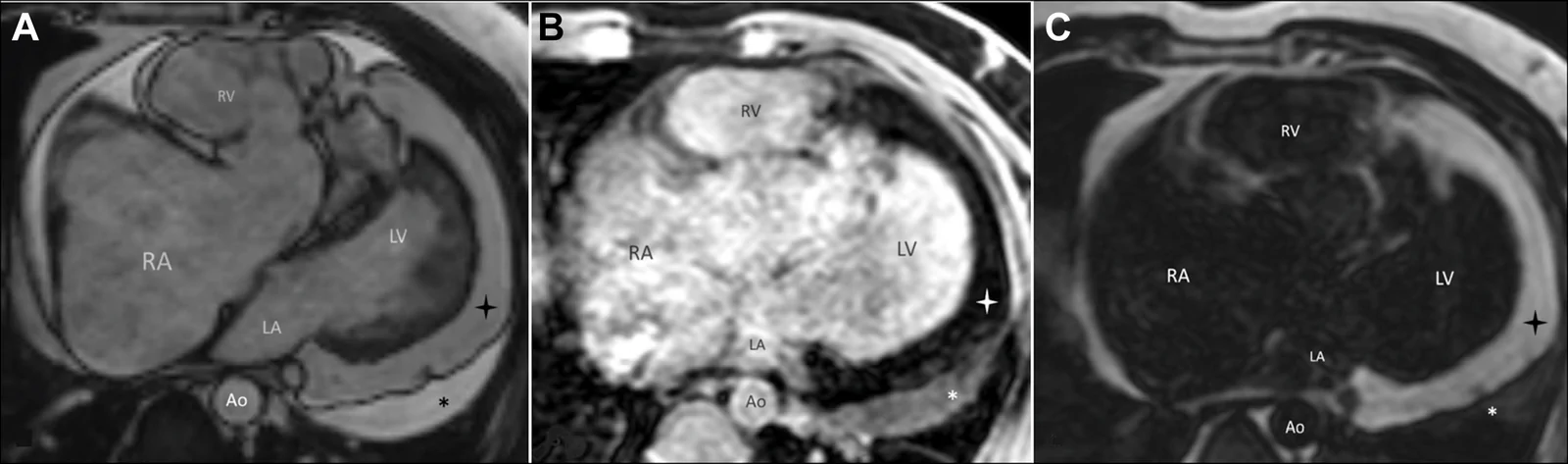
Pericardial effusion and epicardial fat (CMR). **A:** Axial SSFP sequence; Dixon technique, axial view: water (**B**) and fat (**C**) series. The pericardial space exhibits pericardial effusion (shown as ∗), with an estimated separation of pericardial layers of 18 mm, without repercussions on the atria. Likewise, significant lipomatosis of the epicardial fat is observed (shown as ✦), predominantly around the left ventricle, with a maximum thickness of 20.7 mm.

**Figure 5:**
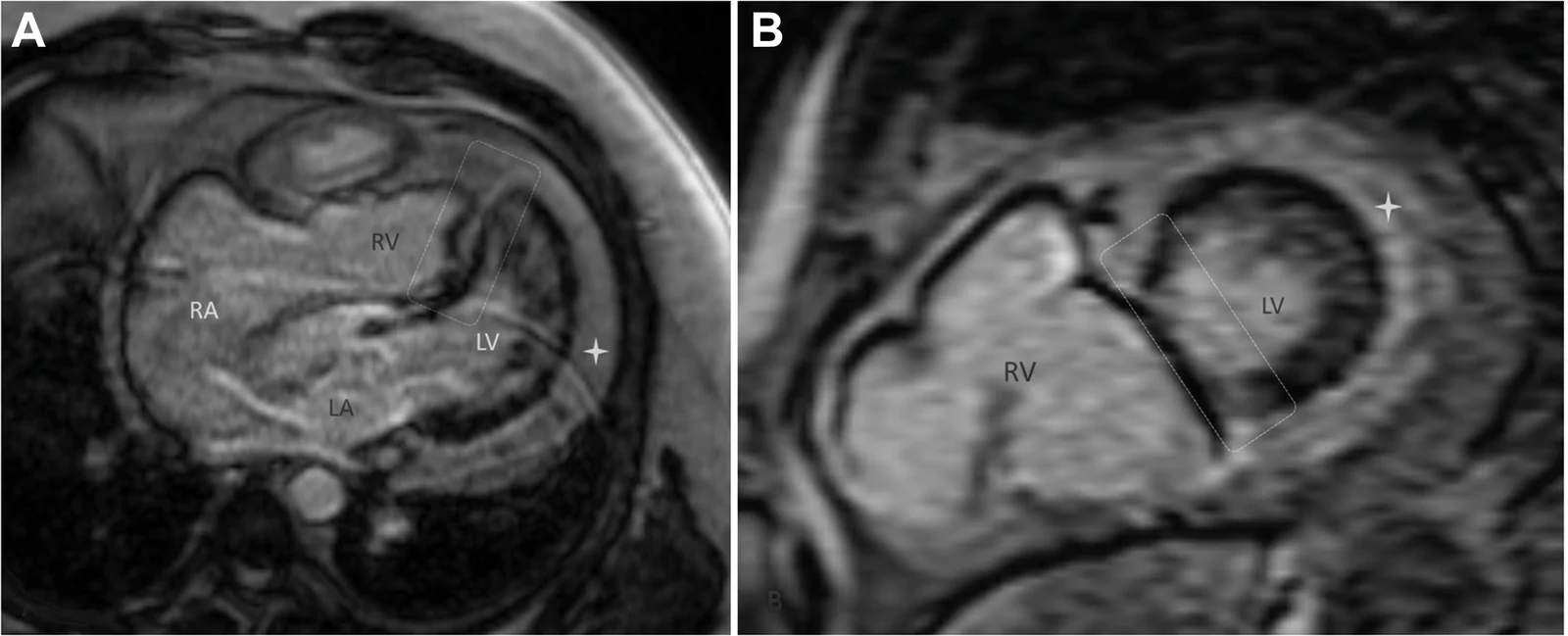
Cardiac magnetic resonance, late gadolinium enhancement. T1-weighted image, late gadolinium enhancement (LGE). **A:** axial view; **B:** short axis, two-chamber view. Late intramyocardial septal enhancement is observed in the basal inferoseptal (segment 3), mid inferoseptal (segment 10), and mid inferior (segment 11). No late enhancement was evident in the right ventricle. ✦: Lipomatosis of epicardial fat.

**Figure 6:**
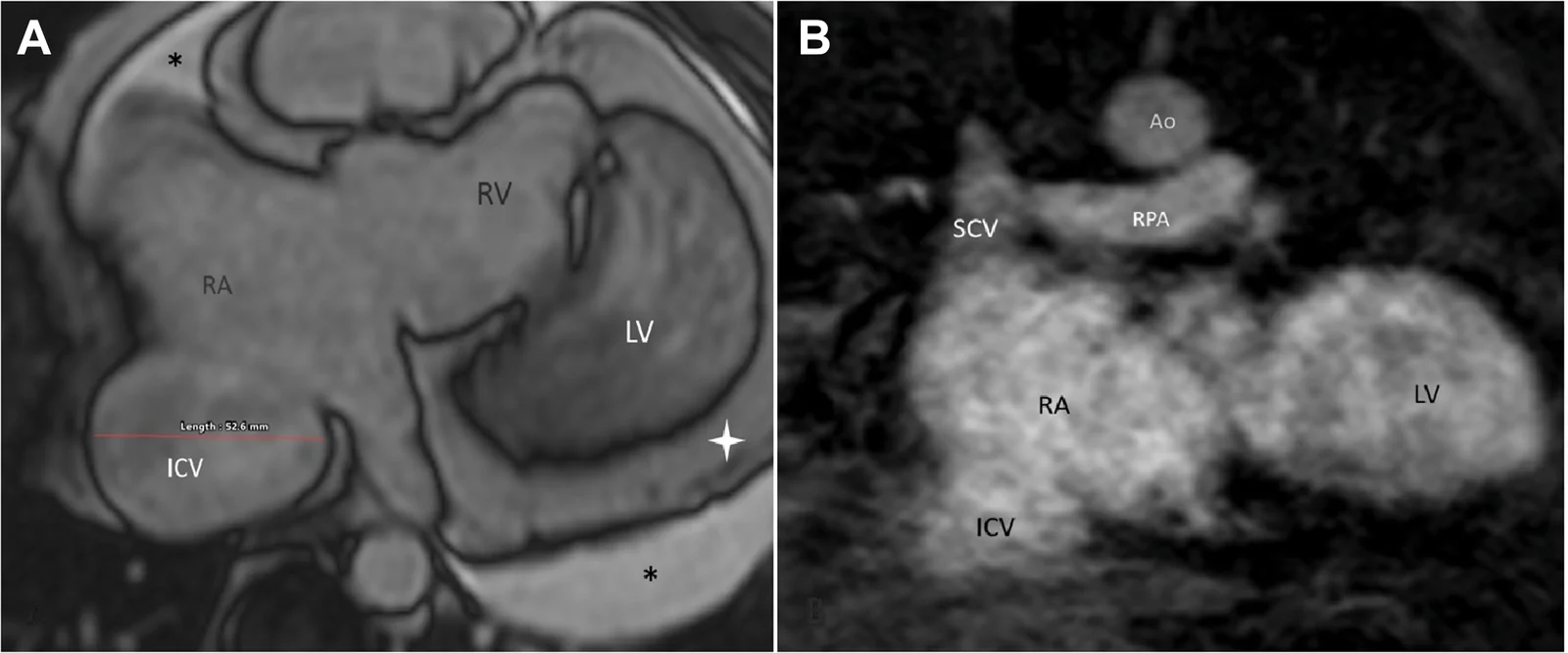
Cardiac magnetic resonance, Venous structures and angiography. **A:** SSFP sequence, axial view; **B:** Tridimensional Contrast-Enhanced Magnetic Resonance Angiography (CE-MRA) sequence, coronal view. This figure demonstrates a moderate enlargement of the inferior vena cava (IVC) shortly before its union with the right atrium (RA). The superior vena cava (SVC) diameter is unremarkable. LV = left ventricle; Ao = Aortic arch; RPA = Right pulmonary artery; ✦ = Lipomatosis of epicardial fat; ∗ = pericardial effusion.

Additionally, a moderate pericardial effusion without hemodynamic compromise and significant lipomatosis of the epicardial fat, predominantly around the LV, were documented (Figure 4). The late gadolinium enhancement (LGE) study showed an intramyocardial non-ischemic uptake pattern in the basal and mid septum of the LV, with no evidence of enhancement in the RV (Figure 5). Furthermore, magnetic resonance angiography showed mild dilation of the inferior vena cava (IVC), with no other relevant thoracic vascular abnormalities (Figure 6).

To assess the arrhythmic profile, 48-hour ambulatory Holter monitoring was performed, which revealed a predominant sinus rhythm with a low burden of premature ventricular contractions (approximately 1000 premature ventricular contractions [PVC] over 24 hours, <1% of total beats), but with arrhythmic complexity, including ventricular couplets and an episode of non-sustained ventricular tachycardia (NSVT). Altogether, these findings suggested ventricular electrical instability, with a dissociation between a low ectopic burden and greater arrhythmic complexity.

As part of the comprehensive evaluation, laboratory studies showed elevated NT-proBNP levels, in the context of normal renal function and electrolytes, which supported the presence of myocardial dysfunction and helped rule out secondary metabolic causes as triggers for the condition.

The integration of clinical, electrocardiographic, arrhythmic, and imaging findings established the diagnosis of ACM. Specifically, the case fulfills definitive diagnostic criteria according to the 2010 Task Force Criteria^12^ by presenting 1 major criterion (RV systolic dysfunction with regional structural alterations) and 2 minor criteria (T-wave inversion in right precordial leads and non-sustained ventricular tachycardia). Concomitantly, the presence of LV involvement characterized by noncompacted myocardium and a non-ischemic LGE pattern allows the case to be accurately classified within the spectrum of biventricular ACM, in accordance with the 2020 Padua criteria.^3^^,^^5^

Given the structural involvement and evidence of ventricular electrical instability, the patient was considered to be at high arrhythmic risk. Guideline-directed medical therapy for heart failure was optimized, including sacubitril/valsartan (25 mg twice a day), dapagliflozin (10 mg once daily [OD]), bisoprolol (1.25 mg OD), and spironolactone (12.5 mg OD). Furthermore, considering the prolonged PR interval, the high arrhythmic risk, and the need to prevent pacing-induced cardiomyopathy, a cardiac resynchronization therapy defibrillator (CRT-D) was implanted. Notably, the procedure used physiologic pacing via left bundle branch area pacing to ensure ventricular synchrony. At the 1-month follow-up, the patient exhibited a highly favorable clinical course, remaining free of presyncope, with no angina and a reported improvement in exercise tolerance. Device interrogation demonstrated successful physiologic pacing without the need for anti-tachycardia pacing or shock therapies during this period.

## Discussion

The present case fulfills the traditional diagnostic criteria for ACM according to the 2010 Task Force Criteria^12^; however, the presence of LV involvement, specifically noncompaction and non-ischemic LGE, supports its classification within the broader spectrum of ACM as defined by the 2020 Padua criteria.^3^^,^^5^ This distinction is clinically critical, as the Padua framework explicitly recognizes biventricular and left-dominant phenotypes that were historically underrepresented and frequently misdiagnosed in earlier models.^3^^,^^5^

It is also important to acknowledge the ongoing nosological debate surrounding LVNC. The American Heart Association classifies LVNC as a distinct primary genetic cardiomyopathy, whereas the European Society of Cardiology has historically categorized it as an “unclassified cardiomyopathy,” considering that hypertrabeculation may represent a morphological trait shared by multiple cardiac diseases rather than a stand-alone entity.^10^ The 2023 European Society of Cardiology Guidelines for the management of cardiomyopathies further refined this position, recommending the descriptive term “left ventricular hypertrabeculation” over LVNC, particularly when the finding is transient or of adult-onset.^11^ This conceptual ambiguity is highly relevant to our case: rather than 2 coexisting independent diseases, the LVNC-like phenotype in our patient may best be understood as part of the structural spectrum of ACM, supporting the unified biventricular ACM diagnosis under the Padua framework.

The coexistence of ACM and LVNC represents a rare but progressively recognized phenotypic overlap. Earlier reports by Tufekcioglu et al^13^, Wlodarska et al^14^, and Aras et al^15^ described patients with isolated ventricular noncompaction mimicking or coexisting with arrhythmogenic RV cardiomyopathy (ARVC), but those reports relied on the 2010 Task Force Criteria, which did not formally accommodate left-dominant or biventricular phenotypes. To our knowledge, the present case is among the first to formally classify ACM-LVNC overlap within the broader biventricular ACM spectrum using the 2020 Padua Criteria, and the first to combine this complex phenotype with cardiac resynchronization defibrillator therapy delivered through physiologic left bundle branch area pacing. This presentation may reflect a shared genetic substrate involving desmosomal proteins. López-Ayala et al^8^ reported a high prevalence of an LVNC-like phenotype in families harboring truncating desmoplakin variants and proposed that LVNC may be 1 of the phenotypic expressions within the broader ACM spectrum, recommending desmoplakin screening in LVNC patients with a high arrhythmic burden. Smith et al^16^ subsequently characterized desmoplakin cardiomyopathy as a distinct fibrotic and inflammatory left-dominant form of ACM.

Emerging evidence suggests that such variants are associated with more extensive myocardial structural changes and a disproportionately high arrhythmic burden, even in the absence of severe global systolic dysfunction.^17^ Although genetic testing could not be performed in this case due to institutional resource constraints, the complex biventricular and noncompaction phenotype strongly points toward this shared genetic etiology. However, from a clinical decision-making standpoint, the severity of the structural phenotype documented by CMR, combined with symptomatic ventricular electrical instability, provides an unequivocal, independent indication for primary prevention device therapy regardless of the specific underlying genotype.

CMR was central to characterizing this complex phenotype, particularly through tissue characterization. The presence of non-ischemic LGE has been consistently associated with life-threatening ventricular arrhythmias and sudden cardiac death, independent of LVEF, and is now recognized as a key arrhythmic risk modifier in ACM.^7^^,^^9^^,^^18^

Cardiac sarcoidosis was carefully considered in the differential diagnosis given the presence of intramyocardial septal late gadolinium enhancement, which is a recognized pattern in this entity. However, several features argued against this diagnosis: the patient’s young age, the complete absence of extracardiac manifestations on detailed systemic review, the absence of high-grade atrioventricular block, and a constellation of structural findings more characteristic of ACM, including RV free-wall sacculations, prominent fibro-fatty changes with marked epicardial lipomatosis, biventricular involvement, and a coexistent LVNC phenotype. We acknowledge that fluorine-18 fluorodeoxyglucose positron emission tomography, the reference modality for ruling out active cardiac sarcoidosis, was not performed because of institutional resource limitations, which represents a limitation. Nevertheless, the integrative phenotypic profile and the absence of any systemic inflammatory features supported the diagnosis of biventricular ACM with overlapping LVNC rather than cardiac sarcoidosis.

Furthermore, this case elegantly illustrates a clinical paradox: a clear dissociation between a very low burden of premature ventricular contractions (<1%) and an increased arrhythmic complexity, manifested as non-sustained ventricular tachycardia. This finding reinforces the limitations of relying solely on quantitative ectopic burden for risk stratification. Instead, it advocates for a multiparametric approach that integrates clinical symptoms, advanced imaging features, and the qualitative arrhythmic profile.^19^ Quantitative arrhythmic risk stratification was further performed using the validated ARVC Risk Calculator developed by Cadrin-Tourigny et al^20^; applying our patients baseline parameters (age 34 years, male sex, no documented cardiac syncope [presyncope only], documented non-sustained ventricular tachycardia, 24-hour PVC count of approximately 1000, 3 anterior leads with T-wave inversion, and RVEF 32%), the estimated 5-year risk of sustained ventricular arrhythmia was 46%. This estimate substantially exceeds the 25%-35% net-clinical-benefit threshold for primary-prevention implantable cardioverter-defibrillator implantation reported in the external validation cohort by Jordà et al,^21^ reinforcing the indication for device therapy.

From a therapeutic standpoint, the combination of arrhythmic symptoms (presyncope), biventricular structural abnormalities, and documented ventricular tachycardia provided a robust rationale for device therapy. Instead of a standard implantable cardioverter-defibrillator, a CRT-D with left bundle branch area pacing was selected. Current international guidelines strongly support device therapy in ACM patients exhibiting these high-risk features, prioritizing the arrhythmic substrate over severely reduced LV function.^22^^,^^23^ Furthermore, the use of physiologic pacing in this case highlights a tailored approach to ensure ventricular synchrony and prevent pacing-induced cardiomyopathy in young patients requiring significant ventricular support.^24^

In accordance with current expert consensus recommendations for inherited arrhythmogenic cardiomyopathies,^22^^,^^25^ cascade clinical screening was offered to first-degree relatives, including detailed family history, resting 12-lead electrocardiogram, and transthoracic echocardiography, with extended evaluation (CMR and ambulatory ECG monitoring) planned for any relative with abnormal initial findings. Genetic counseling was discussed; however, molecular testing of the proband and family members could not be performed due to local resource constraints, and continued longitudinal surveillance is planned in coordination with our institution’s familial cardiomyopathy service.

Ultimately, this case underscores the importance of recognizing complex, overlapping myocardial phenotypes. It reinforces the indispensable role of advanced multimodality imaging and continuous arrhythmic monitoring in identifying vulnerable patients who require early intervention to prevent sudden cardiac death.

## Conclusion

This case highlights a complex presentation of ACM with biventricular involvement and an overlapping LV noncompaction phenotype. It underscores the importance of CMR for accurate phenotypic characterization and risk assessment. Notably, it illustrates that a low ectopic burden does not preclude significant arrhythmic risk, emphasizing the need for a comprehensive, multiparametric approach to guide the timely implementation of advanced device therapy such as a CRT-D with physiologic pacing for the primary prevention of sudden cardiac death.

## Disclosures

The authors report no relationships with industry or other entities relevant to the contents of this manuscript.
